# A clinical study of curative partial breast irradiation for stage I breast cancer using carbon ion radiotherapy

**DOI:** 10.1186/s13014-020-01713-1

**Published:** 2020-11-13

**Authors:** Kumiko Karasawa, Tokuhiko Omatsu, Shintaro Shiba, Daisuke Irie, Masaru Wakatsuki, Shigekazu Fukuda

**Affiliations:** 1 Department of Radiation Oncology, Tokyo Women’s Medical University, 8-1 Kawada-cho, Shinjuku-ku, Tokyo, 162-8666 Japan; 2 National Institutes for Quantum and Radiological Science and Technology, 4-9-1 Anagawa, Inage-ku, Chiba-city, Chiba 263-8555 Japan; 3 Department of Radiation Oncology, Graduate School of Medicine, Gunma University, 3-39-22, Showa-machi, Maebashi City, Gunma 371-8511 Japan

**Keywords:** Carbon ion radiotherapy, Breast cancer, Partial breast irradiation, Non-surgical treatment

## Abstract

**Background and purpose:**

Our institute initiated carbon ion radiotherapy research for patients with stage I breast cancer in April 2013. The purpose of this article is to evaluate the treatment outcome of cases treated outside clinical trial up to May 2020.

**Materials and methods:**

Eligibility criteria of the patients were having untreated stage I breast cancer and being unsuitable for operation for physical or mental reasons. The irradiated volume was defined as the gross tumor including intraductal components. The dose escalation study was initially conducted four times a week for a total of 52.8 Gy [relative biological efficacy (RBE)]. After confirming that adverse effects were within acceptable range, the total dose was increased to 60.0 Gy (RBE).

**Results:**

Between April 2013 and November 2015, 14 cases were treated. The median follow up period was 61 months. No adverse toxicities were observed except for grade 1 acute skin reaction in 10 cases. The time required from carbonion radiotherapy to tumor disappearance was 3 months in 1 case, 6 months in 3 cases, 12 months in 4 cases, and 24 months in 5 cases. The third case developed local recurrence 6 months after radiotherapy. Twelve patients with luminal subtype received 5-year endocrine therapy. Thirteen of 14 tumors have been maintaining complete response with excellent cosmetic results.

**Conclusions:**

The time from carbon ion radiotherapy to tumor disappearance was longer than expected, but complete tumor disappearance was observed except for one high-grade case. With careful patient selection, carbonion radiotherapy in patients with stage I breast cancer is deemed effective and safe, and further research is recommended.

## Introduction

Carbon ion radiotherapy (C-ion RT) has been used for various tumors since our institute initiated clinical trials in 1994, and good results have been reported even for tumors that had been conventionally recognized as radiation-resistant [1–4].

Despite breast cancer being one of the most common cancers in women, clinical trials using C-ion RT for breast cancer had not been conducted prior to 2013. The reason for this was that the role of radiotherapy in breast cancer was regarded as postoperative adjuvant local treatment and therefore the significance of C-ion RT had not be found. Breast cancer treatments are now individualized, taking into consideration various factors. Even for breast irradiation after breast-conserving surgery, options for partial breast irradiation (PBI) as well as whole breast irradiation have now become part of clinical practice [5–7]. In this regard, a clinical trial of curative PBI using C-ion RT was planned for the type of patients that would be eligible for partial breast irradiation by the American Society for Radiation Oncology (ASTRO) consensus statement at that time [8]. Minimally invasive non-surgical treatment is one of the patients’ expectations. C-ion RT is considered suitable for meeting the aim of lessening the treatment burden in early cancer by taking advantage of the high biological effectiveness and better dose distribution.

In 2011, our group had begun preparation to start clinical trials, and in April 2013 we announced the start of a Phase I clinical trial of curative PBI for breast cancer (UMIN ID000010848). The eligibility criteria were low-risk stage I breast cancer, which means pathologically proven invasive ductal carcinoma, Union for International Cancer Control (UICC) T1N0M0, age 60 years and over, estrogen receptor positive, no extensive lymphatic vessel invasion (LVSI), no extensive intraductal component (EIC), and human epidermal growth factor receptor 2 (HER2) negative [9]. This group was considered to be curable by PBI, with no need for whole-breast radiation. After announcing the start of this Phase I study, many patients inquired about applying for the trial, but most did not meet the patient-selection criteria for the clinical Phase I trial, or they did not want tumor resection for pathological evaluation. We advised them to receive standard treatment, but some of them disagreed with our recommendation and expressed eagerness to receive C-ion RT at their own risk. Therefore, we decided to carry out C-ion RT in the framework of “Advanced Medicine” (NIRS ID9401) for patients who could not undergo surgery for medical or mental reasons. Unlike clinical phase trials, patients had to cover some of the medical costs of the C-ion RT. Although the acceptance criteria were relaxed compared to the clinical trial, we did not accept tumors other than stage I or tumors with LVSI or EIC. Treatment was the same as in a forward phase II study of UMIN000010848, which monitored the clinical course without surgical resection of the tumor after C-ion RT. As a result, this decision had an impact on patient accumulation and outcome analysis in the Phase I clinical trial. This article reports the treatment outcome of the 14 “Advanced Medicine” patients.

## Materials and methods

### Eligibility criteria

Eligibility criteria of the patients and tumors were as follows: (1) female; (2) pathologically proven invasive ductal carcinoma of breast; (3) solitary tumor within 2 cm on magnetic resonance image (MRI) including ductal spread, UICC stage I (T1N0M0); (4) no LVSI, no EIC; (5) performance status 0 to 2; (6) life expectancy more than 6 months; (7) those who could not undergo standard treatment for medical or psychological reasons (e.g., having complications difficult to treat anesthetically); (8) those wanting to participate in an “Advanced Medicine” protocol and provide written informed consent. The difference in eligibility criteria from the clinical Phase I trial was the relaxation of age restrictions and the relaxation of restriction of tumor subtype [estrogen receptor (ER) status].

In addition, ineligibility criteria were as follows: (1) having severe complications that could not tolerate the treatment (e.g., uncontrolled cardiopulmonary disease, intractable infectious diseases, uncontrolled mental illness); (2) having a history of treatment for the present breast cancer; (3) being under systemic drug therapy for active double cancer; (4) tumor with chest wall or skin invasion; (5) distance between tumor containing intraductal component and skin less than 5 mm on MRI; (6) having a history of radiotherapy to the expected irradiation site; (7) pathology of non-invasive ductal carcinoma [pure ductal carcinoma in situ (DCIS)]; (8) attending physician considers procedure inappropriate for psychological or other reasons.

Every candidate was the target of careful deliberation concerning eligibility by the breast tumor protocol operational board. Radiation methods of all approved patients were carefully considered and approved at a C-ion RT conference.

### Treatment

C-ion RT was performed using the heavy–ion medical accelerator at our institute. The patient’s fixation was performed with a cast, a breast belt made of elastic fibers in order to compress the contralateral breast and project the affected breast, and a thermoplastic body fixture shell. The prone breast position was not used due to physical limitations of the treatment couch. For position recognition, two fiducial markers were inserted 5 mm from the upper and lower border of the intraductal extension. Biological flatness of the spread-out Bragg peak (SOBP) was normalized by the survival fraction of human salivary gland (HSG) tumor cells at the distal region of the SOBP, where RBE of carbon ions was assumed to be 3.0 [10]. During this period, the passive beam delivery method was adopted. Irradiation was performed using respiratory gating. Irradiation was carried out with a 290 meV/u carbon ion beam via three ports from the front, left, and right of the target. To avoid unnecessary doses to normal tissue, an appropriately sized ridge filter and bolus were selected in each 3 beams. The other details of preparation, positioning, and treatment planning were previously reported [11].

Gross tumor volume (GTV) was defined as the volume of tumor based on contrast MRI findings. Clinical target volume (CTV) was defined as the area of GTV plus intraductal components of the tumor. Planning target volume (PTV) was defined as the region taking into consideration inaccuracies in any geometric variations that may occur in CTV. Dose to the skin was decided as not exceeding 50% of the prescription dose and 30.0 Gy (RBE). Irradiation dose was decided based on the results of clinical trials of 4 times irradiation for stage I lung cancer [12, 13]. The initial fraction dose was 13.2 Gy (RBE), 4 times a week, for a total of 52.8 Gy (RBE). After treating 3 cases, the fraction dose was raised to 15.0 Gy (RBE) and a total dose of 60.0 Gy (RBE).

Following C-ion RT, endocrine therapy was initiated in the patient with Luminal subtype tumor as standard adjuvant treatment and continued for 5 years.

### Treatment evaluation

The primary end point was tumor control, and secondary end points were acute adverse effect, late adverse effect, cosmetic outcome, disease-free survival and overall survival. Acute adverse effects occurring within 90 days from C-ion RT were observed and recorded by NCI Common Terminology Criteria for Adverse Events, Version 4.0 (CTC-AE v4) [14] at the end of treatment, and at 1 month, 3 months and 6 months after. Treatment effect evaluation was performed on MRI and ultrasound (US) images at 1 month, 3 months and once every 6 months after C-ion RT. Late effect was judged by the Radiation Therapy Oncology Group and the European Organization for Research and Treatment of Cancer (RTOG/EORTC) Late Radiation Morbidity Scoring System [15]. Cosmetic outcome was evaluated once every 6 months in terms of breast size, shape, hardness, nipple position compared with the contralateral breast, with grading of poor, fair, good, and excellent.

## Results

Between April 2013 and November 2015, 14 cases were treated. Age of the patients ranged from 44 to 79 years, with a median of 64 years, and tumor size ranged from 9 to 18 mm, with a median of 14.5 mm. ER was positive in 12 patients and negative in 2 patients, and progesterone receptor (PgR) was positive in 7 patients and negative in 7 patients. HER2 was negative in all patients (Table 1). Ki-67 was measured in 8 patients and was found to be over the cut-off value in 3.

**Table 1:** Patient and tumor characteristics

		Number of cases
Age (median)	44–79 years old (64.5)	14
Histology	IDC	14
Subtype	Luminal A	7
	Luminal B	5
	Triple negative	2
Laterality	Right	11
	Left	3
Region	A	5
	AC	3
	C	6
Tumor size (median)	9–18 mm (14)	14

*IDC* invasive ductal carcinoma, *A* upper inner breast, *AC* upper breast, *C* upper outer breast

The first 3 patients were treated with 52.8 Gy (RBE) and had no acute adverse effects except for grade 1 skin reaction in 2 patients. The 3rd patient was 72 years old, with a triple negative subtype and high Ki-67 (60%). She had a history of radical neck dissection and chemotherapy for tongue cancer, and she refused surgery and chemotherapy of any kind. She developed local recurrence and axillary lymph node metastases 6 months after C-ion RT. Since there were no problematic adverse effects in the 3 patients and local recurrence in the 3rd case, we decided on a dose escalation to 60.0 Gy (RBE) from the 4th case.

The only acute adverse effect of C-ion RT in the 14 patients was a grade 1 acute skin reaction in 10 patients.

Endocrine therapy was given to 12 patients for a period of 5 years, consisting of tamoxifen and a luteinizing hormone-releasing hormone (LH-RH) agonist for 2 patients, tamoxifen for 1 patient, and aromatase inhibitor for 9 patients (Table 2).

**Table 2:** Treatment and outcome

		Number of cases
Radiation dose	52.8 Gy (RBE)/4 Fractions	3
	60 Gy (RBE)/4 Fractions	11
Acute skin reaction	Grade 1	10
	Grade 0	4
Follow up period (median)	51–86 months (61)	
Adjuvant therapy	TAM + LHRH	2
	TAM	1
	AI	9
	None	2
Response	CR	13
	PR	1
Recurrence	None	13
	Local + Axillary lymph nod	1

*RBE* relative biological effectiveness, Skin reaction scored by CTCAE ver4, *TAM* Tamoxifen, *LHRH* luteinizing hormone-releasing hormone, *AI* aromatase inhibitor, *CR* complete response, *PR* pathological response

**Fig. 1:**
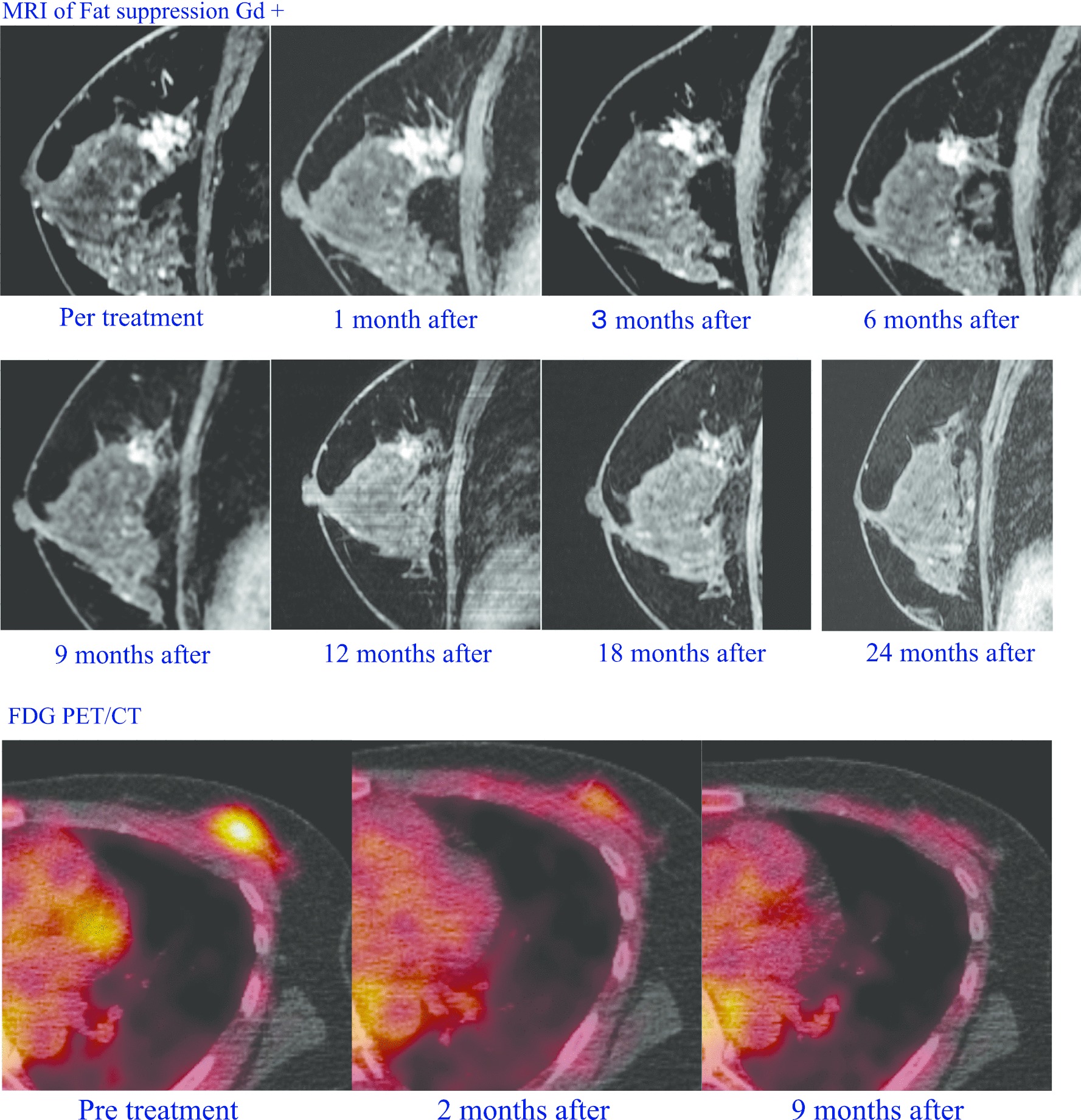
MRI and FDG-PET image of the first case. A 50-year-old female with 20-mm left breast cancer (ER+, PgR-, HER2–) was treated with 52.8 Gy (RBE) C-ion RT and adjuvant 5-year tamoxifen (TAM) and 2-year LH-RH agonist. Contrast-enhanced gradient-echo T1-weighted MR images of pre-treatment, 1 month after, 3 months after, 6 months after, 12 months after and 24 months after C-ion RT. FDG-PET/CT images show the left breast tumor with maximum standardized uptake value (SUV) of 2.5 before treatment. Two months after C-ion RT, FDG uptake was almost normal. At 9 months FDG uptake had disappeared

MRI and US studies were performed 1 month, 3 months, and then every 6 months after C-ion RT in all patients. Figure 1 shows the MRI and FDG-PET images of the first case. At 3 months after C-ion RT, 1 complete response (CR) and 13 partial responses (PR) were observed, at 6 months there were 4 CR, 9 PR and 1 progressive disease (PD) were observed, and at 24 months 13 CR were noted (Table 3).

**Table 3:** Treatment responses on follow-up MRI

Period	1 month	3 months	6 months	12 months	18 months	24 months
Number of cases	14	14	14	13^a^	13^a^	13^a^
CR	0	1	4	8	10	13
PR	6	13	9	5	3	0
SD	8	0	0	0	0	0
PD	0	0	1	0	0	0

*CR* complete response, *PR* pathological response, *SD* stable disease, *PD* progressive disease

^a^One PD case salvaged by surgery is excluded

**Fig. 2:**
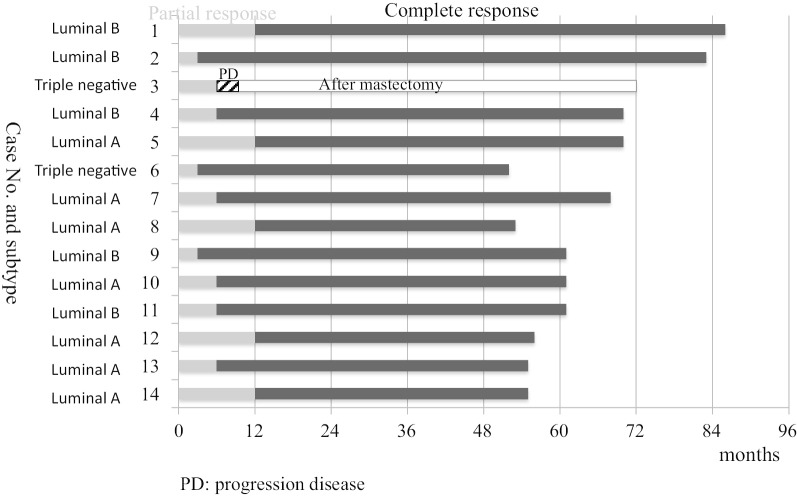
Time to partial response, complete response, and sustainment on follow-up MRI

As for the 3rd patient, who developed local recurrence and axillary lymph node metastases 6 months after C-ion RT, she rejected any type of surgery and chemotherapy at the time of recurrence. Three months following recurrence, however, she underwent a mastectomy and chemotherapy. She continued chemotherapy, but metastases continued to spread, and she died of systemic metastases 69 months after C-ion RT. Figure 2 shows time to partial response, complete response, and sustainment on follow-up MRI by tumor subtype. No difference in these responses was observed depending on the tumor subtype.

As of June 2020, the follow-up period was 51–87 months with a median of 61 months. The other 13 patients survived without recurrence and had no late adverse effect in breast, skin, lung, etc. As for cosmetic outcome, slight pigmentation was observed at the irradiation site and local hardness was increased at the irradiation site up to 3 months after C-ion RT, but there was no difference in breast size, breast shape, or nipple position. Thus, cosmetic outcome was judged to be excellent in all 13 evaluable cases.

## Discussion

In this article, we reported the treatment outcomes of patients with stage I breast cancer who were enrolled in “Advanced Medicine” (NIRS ID9401) between April 2013 and November 2015. Patients not meeting eligibility criteria for the concurrently ongoing Phase I trial (UMIN ID000010848) or refusing to be enrolled in the Phase I trial were registered, so some of them were not in the low-risk breast cancer category. However, these patients were unable to receive standard treatment for physical or mental reasons, and they were accepted into “Advanced Medicine”, except for age and subtype restrictions, from the viewpoint of protecting the patients' right to receive treatment. Of these 14, three patients had comorbidities for which standard surgery was not possible, and one patient had risks with standard surgery. In the other 10 patients, surgery was possible, but their mental status made it unacceptable.

The “Advanced Medicine” program for breast cancer was abolished in March 2016, as C-ion RT for other malignant tumors was covered by national health insurance from April 2016, and the indications for “Advanced Medicine” were narrowed. A Phase II clinical trial of low-risk stage I patients (UMIN ID000010848) and a Phase I trial of stage 0 and intermediate-high risk stage I patients with C-ion RT with standard adjuvant treatment (UMIN ID 000029478) are currently in progress. Only 14 cases were analyzed in the present article because stage I breast cancers registered in “Advanced Medicine” were these 14 cases. Although other clinical trials also have registered stage I breast cancer, we believe it is appropriate to report the results of the trials individually. With a median follow-up of more than 5 years, with the exception of one high-risk recurrent case, 13 patients are alive without breast cancer and have good cosmetic outcomes.

According to MRI, the therapeutic effect of C-ion RT on primary breast tumors appeared slower than expected. As shown in Table 3 and Fig. 2, the time from C-ion RT application to tumor disappearance was 3 months in 1 case, 6 months in 3 cases, 12 months in 4 cases, and 24 months in 5 cases. This result impacted the ongoing Phase I trials. In one Phase I trial, tumor resection was planned 3 months after C-ion RT to assess the pathological effects. However, this assessment 3 months after C-ion RT was then considered premature, based on the results of “Advanced Medicine” patients, and the Phase I study (UMIN ID000010848) of 7 patients was discontinued. Akamatsu et al. [11] had reported the treatment procedure and initial course of the first case, and the subsequent course is shown in Fig. 1. In this patient, it took 24 months for tumor disappearance on MRI, but fluorodeoxyglucose (FDG) positron emission tomography (PET) showed a significant reduction in accumulation at 2 months and complete disappeared at 9 months. In all cases, both early and delayed phase enhancement of the tumor was decrease with tumor shrinkage on dynamic contrast enhanced (DCE) MRI. The accumulation on FDG PET disappeared earlier than the tumor disappearance on MR images in other patients. Assessing the activity was even more difficult by US than by MRI. Then, we recommended performing a needle biopsy on all patients to determine the efficacy of treatment, but all patients refused. The relationship between imaging results and the pathological treatment effects of C-ion RT on breast cancer requires further study. The recommended dose was set at 60.0 Gy (RBE) because no adverse reactions other than Grade 1 acute skin reactions were observed and tumor control was obtained in all cases. Therefore, we have no plans for further dose escalation.

MR images could not identify normal tissue changes within the irradiated area. This may be because the irradiated area is narrow. Breast size, shape, hardness, and nipple position compared with the untreated-side breast did not change at all in any of the cases after a 6-month period. The complete disappearance of the tumor and the absence of normal tissue changes after treatment with MRI and US were the distinguishing points from surgery and other non-surgical treatments. Other non-surgical treatment options for breast cancer usually requiring general anesthesia are associated with inflammatory pain after administration, and the tumor may remain as a mass after treatment. The only invasive procedure for C-ion RT is the insertion of alignment markers with local anesthesia, which is less invasive than other therapies. After 1 to 3 months, the tumor is softened and no longer palpable, so the patient's psychological burden is less than with other treatments. In addition, acute side effects are minimal.

Non-surgical therapies for breast cancer other than C-ion RT include radiofrequency ablation (RFA), cryoablation therapy, high-intensity focused ultrasound (HIFU) and stereotactic body radiotherapy (SBRT). Among them, the most reported is RFA. Ito et al. reported that 386 patients treated with RFA at 10 centers from 2003 to 2009 had a 5-year intra-breast recurrence-free rate of 97% for sizes ≤ 1.0 cm, 94% for 1.1 to 2.0 cm, and 87% for > 2.0 cm or more, respectively [16]. Nguyen et al. [17] reviewed 30 studies, 643 cases of RFA and reported that complete ablation rates ranged from 100 to 44% with a median of 88%. In cryoablation therapy, Lanza et al. [18] reviewed 7 studies, 176 cases performed from 2003 to 2013, and found that complete local tumor control was 73%. In the HIFU, She et al. reported that tumor residual rates in 6 studies for breast cancer ranged from 0 to 90% [19]. Regarding radical SBRT, there are even fewer reports. Shibamoto et al. reported that they performed whole breast irradiation and SBRT boost with the radiosensitizer KORTUC [20] for 18 patients who refused surgery, and only one case developed local recurrence [21]. Barry et al. [22] reviewed SBRT for breasts and reported that five neoadjuvant setting phase I/II trials were underway, including the ARTEMIS trial in Canada and the ABLATIVE trial in the Netherlands. In the ABLATIVE trial, thirty-six patients were treated with neoadjuvant partial breast irradiation, and pathological complete response (pCR) was reported in 42% patients after an interval of 6 to 8 months with transient grade 2 and 3 toxicity in 31% and 3% of patients [23]. Compared with these treatment results, C-ion RT is presumed to have merits of high tumor control and low adverse events.

As far as we know, reports of clinical studies of C-ion RT for breast cancer are limited to our institute. Basic research has been reported from various institutes [24–27], indicating that they are indeed interested in C-ion RT for breast cancer. With the growing number of C-ion RT facilities in the world, we hope that other facilities will actively research curative C-ion RT for breast cancer. Scanning and rotating gantry for respiratory movements are available at our facility, and the ongoing clinical trials use scanning irradiation to obtain better dose distribution.

At present, because of the cost of treatment and the limited number of facilities, C-ion RT for breast cancer is a limited research treatment, but research on miniaturization of device and price reduction is progressing [28]. We believe that clinical research of C-ion RT for breast cancer must be continued in preparation for the day when this treatment will become accessible to many patients.

## Conclusions

The time from C-ion RT to tumor disappearance was longer than expected, but complete tumor disappearance was observed except for one high-grade case. With careful patient selection, C-ion RT in patients with stage I breast cancer is deemed effective and safe. We think it is worth continuing further research.

## Data Availability

The datasets used and/or analyzed during the current study are available from the corresponding author on reasonable request.

## Abbreviations

RBE: Relative Biological Efficacy
C-ion RT: Carbon ion radiotherapy
PBI: Partial breast irradiation
ASTRO: American Society for Radiation Oncology
UMIN: University hospital Medical Information Network
UICC: Union for International Cancer Control
LVSI: Lymphatic vessel invasion
EIC: Extensive intraductal component
HER2: Human epidermal growth factor receptor 2
NIRS: National Institute of Radiological Sciences
MRI: Magnetic resonance image
ER: Estrogen receptor
DCIS: Ductal carcinoma in situ
GTV: Gross tumor volume
CTV: Clinical target volume
PTV: Planning target volume
MeV: Mega electron volt
CTC-AE: Common Terminology Criteria for Adverse Events
US: Ultrasound
RTOG/EORTC: Radiation Therapy Oncology Group and the European Organization for Research and Treatment of Cancer
PgR: Progesterone receptor
LH-RH: Luteinizing hormone-releasing hormone
CR: Complete response
PR: Partial responses
PD: Progressive disease
FDG: Fluorodeoxyglucose
PET: Positron emission tomography
DCE: Dynamic contrast enhanced
